# The Impact of Informational Intervention on HPV Vaccination Intention among Heterosexual Men

**DOI:** 10.3390/vaccines11111653

**Published:** 2023-10-27

**Authors:** Songyang Zhang, Leigh H. Grant, Janet Geipel, Zhihan Cui, Boaz Keysar

**Affiliations:** 1 Department of Psychology, University of Chicago, Chicago, IL 60637, USA; songyangzhang@uchicago.edu (S.Z.); burnettlh@uchicago.edu (L.H.G.); 2 Department of Business Strategy and Marketing, University of Exeter Business School, Exeter EX4 4PU, UK; 3 Anderson School of Management, University of California, Los Angeles, CA 90095, USA; zhihan.cui@anderson.ucla.edu

**Keywords:** human papillomavirus (HPV), vaccine hesitancy, informational intervention, health psychology

## Abstract

Human papillomavirus (HPV) is one of the most common sexually transmitted infections (STIs). However, despite widespread under-vaccination amongst men and the importance of vaccinating both sexes to curb the spread of HPV, research has focused on promoting HPV vaccination predominantly amongst women. Therefore, the current study examines the effectiveness of different informational interventions in promoting vaccination intentions amongst heterosexual men. In a preregistered study of 583 unvaccinated adult men, we randomly assigned participants to one of four informational interventions aimed at promoting awareness of HPV risks and vaccine uptake: (1) risks to oneself (*n* = 145), (2) risks to their female partner (*n* = 144), (3) risks to oneself and their female partner (*n* = 153), and (4) general vaccine information (*n* = 153). Amongst participants reporting a sexual history (67%), intentions to get vaccinated significantly increased by 10.75 points on a 100-point scale (*p* < 0.01) after they received information about the risks of HPV for both themselves and their female partner, compared to receiving information about only their own HPV risk. These findings provide valuable guidance for public health officials and policymakers into the effectiveness of different messaging strategies in promoting HPV vaccination amongst adult male populations to increase vaccination rates.

## 1. Introduction

Human papillomavirus (HPV) is one of the most common sexually transmitted infections (STIs) worldwide. Currently, over 43 million Americans are infected with HPV, and an estimated 13 million Americans become infected each year [1,2]. While most HPV infections resolve themselves without further health complications, high-risk HPV types can cause cervical, vaginal, and vulval cancer in women, as well as anal and penile cancer in men [3,4]. Currently, getting vaccinated against HPV is a highly effective and low-risk preventative option that can reduce the likelihood of developing HPV by as much as 90% [5,6]. Furthermore, HPV vaccination has the potential to reduce the need for cancer screening, thereby reducing health care costs [7]. Because of its effectiveness, the Center for Disease Control (CDC) currently recommends all young adults below the age of 27 get the HPV vaccine [2]. Although the HPV vaccine is most effective when received before the initiation of sexual activity [8], it can also prevent new HPV infections in individuals who have already become sexually active [9]. Therefore, catch-up HPV vaccination is recommended for all persons through age 26 regardless of prior sexual history [9].

Despite the effectiveness and availability of the HPV vaccine, uptake remains low, particularly among eligible men. Among eligible men, only 27.0% have opted to receive at least one dose of the HPV vaccine, and among those only a dismal 9.0% ended up completing all the recommended doses. In contrast, among eligible women, 53.6% have received at least one dose of the HPV vaccine, with 27.0% completing all the recommended doses [10]. Therefore, finding effective ways to promote HPV vaccination uptake, particularly amongst men, is essential to curbing the spread of HPV infection. One possible low-cost method is informational interventions, which aim to increase vaccination by increasing awareness and knowledge of HPV and the HPV vaccine (for a meta-analysis, see [11]).

While informational interventions have been found to be effective in promoting HPV vaccine uptake, these interventions have focused on adult women [12]. In a systematic review of 35 studies on interventions to improve HPV vaccination coverage, 22% of studies tested adult female populations. In contrast, only 3% tested adult male populations (all other studies tested parents, health care providers, or a mix of parents and adolescents) [12]. Therefore, most studies have failed to consider HPV vaccination uptake amongst adult men. Furthermore, even fewer studies have addressed what types of male-specific informational interventions may be most effective in promoting HPV vaccination intentions in this population.

The current research evaluated the effectiveness of different informational interventions in promoting HPV vaccination intentions for adult heterosexual men. We achieved this by experimentally investigating the effectiveness of different information interventions that highlight the risk of HPV for themselves, their female partner, or both themselves and their female partner. Our rationale for these different informational interventions is linked to the sex-specific nature of certain cancer types linked to HPV. Because some cancers linked to HPV are male-specific (penile cancer), female-specific (cervical, vaginal, and vulval cancer), or apply to both sexes (anal and throat cancer), understanding whether it is important to highlight the cancer risks of HPV for oneself, one’s partner, or both is of theoretical interest. Following the informational intervention, we measured HPV vaccination intentions in each condition.

To date, only a limited number of studies have addressed the extent to which self- and partner-oriented informational interventions influence HPV vaccination. These studies have yielded mixed results. While one study suggested that both self- and partner-oriented HPV risk information can be more effective than general HPV risk information in promoting HPV vaccine acceptance amongst heterosexual adults [13], other studies did not detect such a difference [14]. For instance, informing college students about the benefits of male HPV vaccination for reducing cancer risk in women and men, as compared to the benefits for only men, did not significantly increase young men’s interest in the HPV vaccine [13]. Another study provided evidence that informational interventions can significantly increase intentions to get vaccinated against HPV within the next 12 months among male participants [15]. However, this effect was independent of whether participants were informed about the benefits for women (preventing cervical cancer), personal sexual protection (preventing genital warts), or personal cancer protection (preventing head and neck cancer). Importantly, though, these studies come with two major limitations. First, they largely examined vaccination intentions among a smaller, university-based male population, and hence results to date may have been specific to the subpopulation being studied. Additionally, in the case of null results, these studies may have failed to detect a significant effect simply because the number of participants tested was too small to reliably detect intervention effects.

In light of the limited research on male-specific HPV vaccine interventions, the current randomized experiment aimed to systematically examine the impact of providing HPV risks for oneself as compared to one’s female partner on HPV vaccination intentions among heterosexual adult men. To do so, we employed a full randomized experimental design, testing the impact of an informational intervention that highlights the HPV risks to oneself (Self), the HPV risks to one’s female partner (Partner), the HPV risks to both oneself and one’s partner (Both: Self and Partner), and a condition that highlights neither the risk to oneself nor to one’s partner (Control). From this experimental design, we can test two competing theories for the impact of self-oriented or partner-oriented informational interventions on HPV vaccine intentions of male participants.

On the one hand, partner-oriented HPV risk information may be less effective in boosting vaccination rates compared to offering information that is more directly self-relevant. This is because men tend to be less communally oriented than their female counterparts [16]. Hence, messaging that highlights how vaccination may benefit another person may be less effective in incentivizing vaccination behavior than messaging that highlights the benefits to oneself. This theory predicts that self-oriented messaging would be more effective than partner-oriented messaging in boosting HPV vaccination intentions, with both self- and partner-oriented messaging performing either comparably or worse than self-oriented messaging. Alternatively, it may be that partner-oriented messaging is more effective in promoting HPV vaccination intentions beyond simply offering information about oneself. This is because men tend to be less likely to participate in preventative health behaviors [17], primarily since acting in a preventative, self-protective manner can be seen as violating masculine norms among heterosexual men [18]. Therefore, highlighting how HPV vaccination instead benefits a sexual partner may act as a salient benefit without violating masculine norms, thereby promoting vaccination more effectively than just highlighting the benefits of vaccination for oneself. This theory predicts that partner-oriented messaging would outperform self-oriented messaging in boosting HPV vaccination intentions, with both self- and partner-oriented messaging performing better than only self-oriented messaging.

In sum, our objective was to experimentally assess whether including partner-oriented risk information increases HPV vaccination intentions among adult heterosexual men. In doing so, we provide valuable insight for public health officials into what informational intervention methods may be most effective in boosting HPV vaccination amongst an under-vaccinated and often understudied male population.

## 2. Materials and Methods

### 2.1. Ethics and Registration

This research was approved by the University of Chicago Institutional Review Board (IRB21-2010). The study design, including the number of participants, number of conditions, exclusion criteria, and planned analyses were preregistered on the Open Science Framework [19].

### 2.2. Study Design

We conducted a randomized experiment in which we randomly assigned participants of a convenient online sample to one of four HPV vaccine informational interventions (Self, Partner, Both: Self and Partner, Control).

### 2.3. Power Analysis

An a priori calculation of the required sample size, anticipating a medium sized effect (Cohen’s *f* = 0.15) with four experimental groups at a power of 95%, yielded a total sample size of 580 participants (145 participants per condition). We therefore preregistered 580 participants (after exclusion). We anticipated ~5% exclusions (based on previous studies using online samples) and therefore requested slightly more online participants.

### 2.4. Sample Preparation

Participants were first prescreened to ensure they (1) were heterosexual males between 18 and 26 years old currently residing in the United States, (2) were eligible to receive the HPV vaccine, (3) had not been previously vaccinated or currently had an appointment to get vaccinated against HPV (“Have you already received at least one dose of the human papillomavirus [HPV] vaccine” and “Are you currently scheduled to receive an HPV vaccination?”, No, Yes, Unsure; Only participants who answered “No” for both questions were eligible to participate), and (4) currently had health insurance. Eligible participants were invited to participate in the main online experiment. After consenting to participate, each eligible participant was randomly assigned to one of four informational intervention conditions. See Appendix A, Table A1 for the exact wording of the prescreen measures. As mentioned in 2.3, to ensure at least 580 participants after exclusion, we recruited 606 participants in April 2023 online through Prolific (www.prolific.co, accessed on 1 April 2023) and CloudResearch (www.cloudresearch.com, accessed on 7 April 2023). Of these, 23 participants (3.8%) were excluded according to our preregistered exclusion criteria. Participants were excluded if they did not pass the attention check (*n* = 7), indicated that they did not pay attention to the intervention messages (*n* = 8), or failed to provide any relevant information regarding HPV or the HPV vaccine in the open response question (*n* = 8).

### 2.5. Participants

The online convenience sample consisted of 583 heterosexual adult males living in the U.S. (*M_Age_* = 22.48 years, *SD* = 2.46, age range: 18 to 26 years). We tested adults aged 18 to 26 with and without a prior history of sexual activity because HPV vaccination is recommended for all individuals up to the age of 26 regardless of their sexual history [9]. Participants were randomly assigned to one of four HPV risk information conditions: Self (*n* = 145), Partner (*n* = 144), Both (*n* = 141), or Control (*n* = 153). A one-way between-subject analysis of variance (ANOVA) with 583 participants across four conditions would be sensitive to effects of Cohen’s *f* = 0.17 (Cohen’s *d* = 0.34) with 95% power (α = 0.05, two-tailed). This means the study would not be able to reliably detect intervention effects smaller than Cohen’s *f* = 0.17 (Cohen’s *d* = 0.34).

### 2.6. Intervention

In the US, universities and colleges recommend but rarely mandate HPV vaccination upon college entry [20]. Therefore, informational interventions could make an important contribution to promoting HPV vaccination uptake. To evaluate such interventions, our participants were presented with parts of the vaccine information sheet which was developed and used by the Center for Disease Control and Prevention [2] (www.cdc.gov/std/hpv/stdfact-hpv.htm, accessed on). We presented participants with this information because empirical evidence about messaging campaigns suggests that providing information about HPV is effective in boosting HPV vaccination intention [21]. The vaccine sheet provides a comprehensive set of information about HPV infection, HPV vaccines, and HPV-associated male and female diseases. First, participants saw the following information about the HPV infection:

HPV (Human Papillomavirus):

HPV infections are very common. Nearly everyone will get HPV at some point in their lives. Currently, more than 42 million Americans are infected with HPV types that cause disease. About 13 million Americans, including teens, become infected each year. HPV is spread through intimate skin-to-skin contact. You can get HPV by having vaginal, anal, or oral sex with someone who has the virus, even if they do not have signs or symptoms. Most HPV infections (9 out of 10) will go away on their own within 2 years. But sometimes, HPV infections will last longer and can cause some varieties of cancer. Every year in the United States, HPV causes about 36,000 cases of cancer in men and women.

Then, participants saw the following information about the HPV vaccine:

HPV Vaccination Information:

The HPV vaccine provides safe, effective, and long-lasting protection against cancers caused by HPV. The HPV vaccine can prevent over 90% of cancers caused by HPV. More than 135 million doses of the HPV vaccine have been distributed since they were licensed in 2006. Data continue to show the vaccines are safe and effective. People who get the first dose at or after 15 years of age need 3 doses of the HPV vaccine. The recommended three-dose schedule is to get the initial dose, then the second dose 1–2 months later, and finally the third dose 6 months after the initial dose.

Next, participants saw one of four messages containing a call-to-action line encouraging participants to get vaccinated against HPV. The Self condition stated “Protect yourself. Get vaccinated today”, the Partner condition “Protect your partner. Get vaccinated today”, and the Both condition “Protect yourself and your partner. Get vaccinated against HPV today”. The Control condition stated “Get vaccinated against HPV today”.

Following the call-to-action line, all participants saw information about different cancer types that are associated with HPV infection and for which the vaccine can reduce the risk of development. The content of each intervention is briefly summarized in Table 1 (the full text of each intervention can be found in Appendix A, Table A2). The main distinctions between the different intervention information were the different types of cancer that HPV infection may cause in female or male populations [22]. In an assessment of HPV infections in the U.S., research estimated the impact of HPV infection on causing specific types of cancer for both female and male populations [22]. Developed from the statistics and information from the assessment, the Partner condition included HPV-associated female diseases such as anal, cervical, vulval, and oropharyngeal cancer; the Self condition included HPV-associated male diseases such as anal, penile, and oropharyngeal cancer. The Both condition included HPV-associated male and female diseases. All intervention conditions (Self, Partner, Both) included general information about the vaccine that was presented in the control condition.

The different parts of the information messages (i.e., HPV, HPV vaccine, HPV-related cancer) for all intervention conditions and the Control condition were shown on single pages with an embedded tool that prevented participants from proceeding to the next page for a set duration of time. This was implemented to prevent participants from rushing through the experimental materials. Because of the differences in length of the informational intervention conditions, the minimum amounts of time participants were required to stay on the informational page varied depending on condition: 60 s for the Both condition, 45 s for the Self and Partner conditions, and 30 s for the Control condition. While participants could continue once their minimum time passed, they could stay on the page as long as they needed. To ensure participants read the intervention information, we included an open response question asking participants to report information mentioned in the intervention message. Additionally, we included an attention check question at the end of the experiment (i.e., “Please tell us honestly: did you pay attention to the HPV vaccine information at the beginning of the survey? Your answer will not affect your payment.”; 1 = Yes I paid attention, 2 = No I did not pay attention). Participants who failed to provide any information to the open response question or reported not paying attention were excluded.

### 2.7. Outcome Measure

Following the vaccine messages, participants were asked to report their intentions to get vaccinated against HPV (“On a scale from 0% [definitely will not] to 100% [definitely will], what is the likelihood that you will get the HPV vaccine in the next 3 months?”). Following the intention measure, participants completed four additional blocks of questions to capture the following: their trust in the shared information, safety, and effectiveness of the vaccine; their attitudes regarding HPV and the HPV vaccine; the perceived social norm of getting vaccinated; and finally their perceived behavioral control in getting the HPV vaccine. To avoid order effects, these blocks were presented in a randomized order across participants.

Beginning with the trust measures, participants reported their trust in the information provided as well as the perceived safety and effectiveness of the HPV vaccine, with all three items captured on a scale from 1 (do not trust at all) to 6 (completely trust). To capture attitudes about HPV and the HPV vaccine, participants answered a series of questions including how worried they were about getting the HPV vaccine (on a scale from 1 [not at all worried] to 5 [extremely worried]), how beneficial they perceived the HPV vaccine as being (on a scale from 0 [not at all beneficial] to 100 [extremely beneficial]), how risky they perceived the HPV vaccine as being (on a scale from 0 [not at all risky] to 100 [extremely risky]), and finally how seriously they perceived the possible side effects of the HPV vaccine and a possible HPV infection as being (on a scale from 1 [not at all serious] to 5 [extremely serious]).

Next, to capture perceived social norms of getting vaccinated, participants were asked a series of three questions assessing the extent to which they believed people expected them to get vaccinated. These included statements such as “It is expected that I will get vaccinated against HPV”. All items in this set were rated on a scale from 1 (completely disagree) to 7 (completely agree). Finally, to capture perceived behavioral control, participants responded to three questions asking them to report whether getting vaccinated against HPV was something that they had control over, such as “I am confident that I can get the HPV vaccine”. All items in this set were rated on a scale from 1 (strongly agree) to 7 (strongly disagree).

Then, the participants were asked to select “5” on the Likert scale from 1 to 7 as the first attention check question. The participants were asked “How confident are you that you understood the HPV information”, rated on a scale from 1 (very confident) to 7 (extremely confident). Furthermore, they were asked to explain their decision rationale for their likelihood to get HPV vaccination in the next three months in the text box.

At the end of the main study measures, participants completed two scales to capture both pre-existing attitudes around vaccinations as well as the extent to which they identified as being aligned with agentic or communal values. First, the Attitudes Toward Vaccines scale [23] measured general vaccine attitudes, with items asking participants to report the extent to which they agree with statements such as “Doctors give out too many vaccines” or “Vaccines are a good way to protect public health”. Following each of the eight statements, participants reported their agreement on a scale from 1 (strongly disagree) to 6 (strongly agree). Then, participants completed the Agentic and Communal Values scale [24], in which participants reported the importance of 24 different values (such as Wealth, Pleasure, Forgiveness, or Influence) on a scale from 1 (not quite important to me) to 9 (highly important to me).

Prior to completing the experiment, demographic information was collected. This included information on each participant’s sexual history (“Have you ever had sex [including vaginal, oral, anal sex]?” Yes, No, Prefer not to say), current sexual activity, number of sexual partners, current relationship status, and relationship exclusiveness. Participants also provided general demographic information on their current level of education, employment status, household income, occupation, age, political affiliation, and ethnicity (see Table 2 for response options and the Appendix A, Appendix B and Appendix C for the exact wordings of these measures). We further asked participants to report their attention while completing the experiment (see details above). Finally, we measured a secondary outcome variable, whether participants clicked a link to look up nearby pharmacies that carry the HPV vaccine. For this, participants read the following message at the end of the study: “Thank you so much for your participation. If you are interested in getting vaccinated against HPV, please visit the link below to find a pharmacy near you that carries the HPV vaccine. [Link Text: Find a Pharmacy Near You]” (1 = clicked, 0 = not clicked).

### 2.8. Statistical Analysis

#### 2.8.1. Intervention Effects on HPV Vaccine Intentions

We first conducted ANOVA analysis to evaluate if HPV vaccination intention differed significantly across the four conditions. Moreover, to assess the impact of interventions on HPV vaccination intentions—as well as on the secondary measures including attitudes, social norms, and behavioral control—a series of linear regression models with planned contrast analyses were conducted. These models assessed three comparisons which tested (1) the effectiveness of highlighting the risks of HPV to themselves or others as compared to only offering more general information on HPV and the HPV vaccine (Self, Partner, and Both vs. Control), (2) the effectiveness of highlighting self- as compared to partner-oriented risks of HPV (Self vs. Partner), and finally (3) the effectiveness of highlighting both self- and partner-oriented risks of HPV as compared to only presenting information on the risks to oneself (Both vs. Self). We conducted one planned contrast analysis comparing the difference in HPV vaccination intention and another comparing the difference in HPV vaccination intention while controlling for variance in demographic characteristics (age, education, ethnicity, income, occupation, and relationship and sexual history). Furthermore, to measure vaccination intentions, we initially planned to examine reported vaccination intentions as well as clickthrough rates in which individuals searched for locations in which the HPV vaccine was available using a link at the end of the study. However, due to low clickthrough rates in the current study (1.54%), this measure was dropped from the analysis.

#### 2.8.2. Factors Associated with HPV Vaccination Intentions

Along with examining the impact of each intervention on vaccination intentions, we conducted a series of exploratory moderation analyses to examine whether the influence of each informational intervention varied depending on the characteristics of the individual receiving the message. To test this, we examined whether relationship status and sexual history, general attitudes regarding vaccines, and relative endorsement of agentic as compared to communal values interacted with the impact of informational intervention on vaccination intentions.

#### 2.8.3. Relationship Status and Sexual History

Additionally, we examined whether variance in relationship and sexual history influenced how participants responded to the informational interventions. Because HPV is a sexually transmitted disease, people who have more limited relationship and sexual histories may feel less compelled to get vaccinated overall, as they may find the informational interventions to be less self-relevant. To test this theory, we conducted a series of separate planned contrast analyses including both the main effect and interaction of sexual history, current sexual activity, and relationship status on vaccination intentions across informational interventions.

#### 2.8.4. Attitudes, Perceived Norms, and Perceived Behavioral Control

Finally, we examined whether attitudes, perceived norms, and perceived behavioral control mediated the effect of informational interventions on HPV vaccination intention. Prior to running the mediation analyses and consistent with the Baron and Kenny [25] method of mediation analysis, we first checked whether each factor met the conditions for mediation. However, none of the factors yielded a significant difference in ratings across messaging conditions, and hence violated the requirements for mediation (see Appendix B, Table A3 for the means, standard deviations, and significance level of each planned contrast for further details).

## 3. Results

### 3.1. Sample Characteristics

The descriptive demographic characteristics for individuals demonstrate that the four conditions were similar in terms of demographic traits. All participants were heterosexual men between the ages of 18 and 26 years old. A detailed report of the demographic statistics by condition is shown in Table 2.

### 3.2. Intervention Effects on HPV Vaccination Intentions

**Figure 1:**
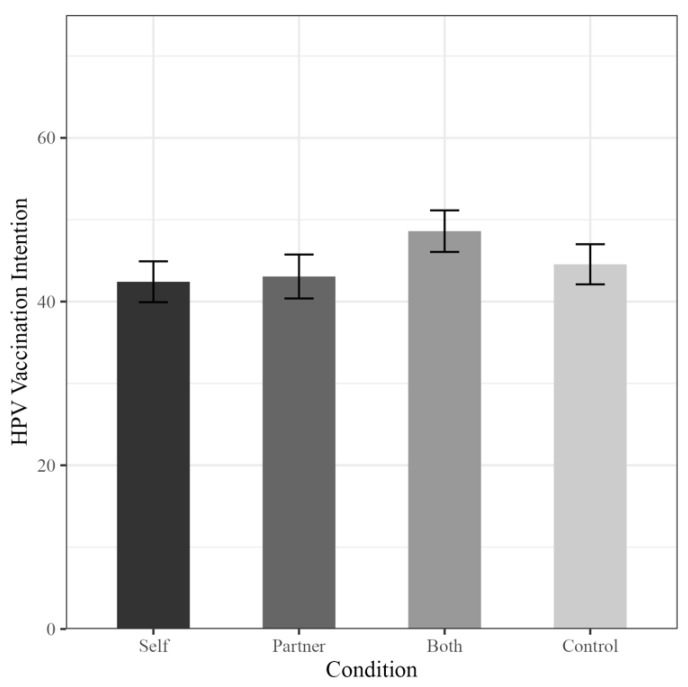
Male HPV vaccination intention by condition. Note: *y*-axis truncated for clarity. Errors bars illustrate standard errors of the means.

Overall, the four intervention conditions did not differ significantly from each other (Self: *M* = 42.42, *SD* = 30.05, 95% CI [37.53, 47.31]; Partner: *M* = 43.06, *SD* = 32.21, 95% CI [37.80, 48.32]; Both: *M* = 48.60, *SD* = 30.15, 95% CI [44.62, 53.57]; Control: *M* = 44.56, *SD* = 30.31, 95% CI [39.76, 49.36]; *F* [3, 579] = 1.16, *p* = 0.32) (See Figure 1). Furthermore, this pattern of results held when including demographic characteristics as covariates in the model (*F* [3, 530] = 1.46, *p* = 0.23).

However, among the three intervention conditions (Both, Partner, and Self), an interesting pattern emerged. When controlling for demographic characteristics of the sample (see details above), participants in the Both condition reported significantly higher HPV vaccination intention than participants in the Self condition (Both vs. Self: *b* = 4.21, *t* [530] = 1.97, *p* = 0.05, 95% CI [0.01, 8.41]). There were no significant differences in vaccine intentions between participants in the Self condition and participants in the Partner condition, both with and without controls for demographic characteristics (Self vs. Partner, without demographics: *b* = 1.63, *t* [579] = 0.78, *p* = 0.44, 95% CI [−5.74, 2.47]; with demographics: *b* = 1.70, *t* [530] = 0.81, *p* = 0.42, 95% CI [−5.83, 2.43). There was also no significant difference in vaccine intentions between participants in the Both condition and participants in the Self condition if demographic factors were not accounted for (Both vs. Self; *b* = 3.91, *t* [579] = 1.86, *p* = 0.06, 95% CI [−0.22, 8.04]).

### 3.3. Sexual History and Relationship Status

Sexual history had a significant main effect on intentions to get vaccinated (*b* = 12.62, *t* [549] = 4.50, *p* < 0.001, 95% CI [7.11, 18.12]). When including sexual history (yes, no, omitting the “prefer not to say” responses) as a covariate in the model, participants in the Both condition reported significantly higher HPV vaccination intentions than participants in the Self condition (Both vs. Self; *b* = 10.75, *t* [549] = 2.30, *p* < 0.05, 95% CI [1.57, 19.93]). However, participants of all intervention conditions reported similar vaccination intentions as participants in the Control condition (Interventions vs. Control; *b* = 0.35, *t* [549] = 0.41, *p* = 0.68, 95% CI [−2.03, 1.32]). Also, participants in the Partner condition reported similar intentions to vaccinate to participants in the Self condition (Partner vs. Self; *b* = 2.37, *t* [549] = 0.94, *p* < 0.35, 95% CI [−7.33, 2.58]).

**Figure 2:**
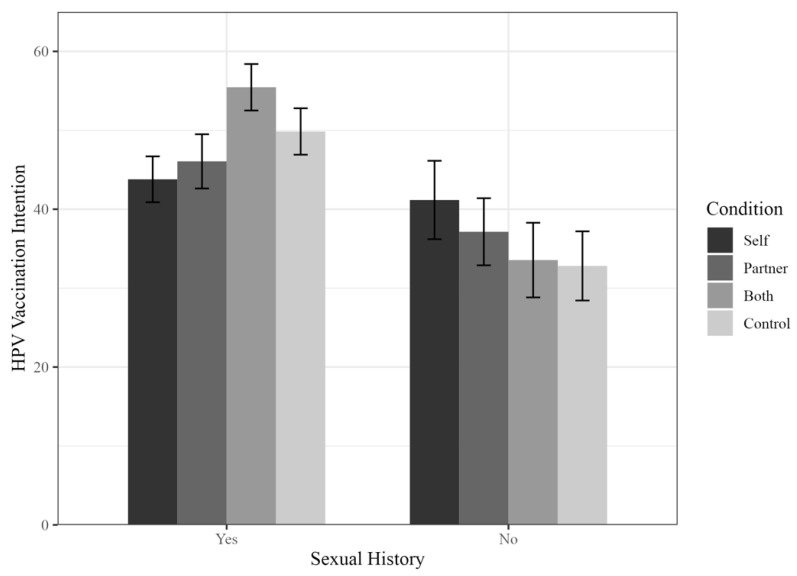
Impact of condition on male HPV vaccination intention as a function of sexual history. Note: *y*-axis truncated for clarity. Error bars illustrate standard errors of the means.

Sexual history (yes, no, omitting “prefer not to say” responses) also significantly interacted with intervention conditions (Both vs. Self) (*t* [549] = 2.30, *p* < 0.05). Here, participants who had sexual experiences (67.2%) in the Both condition (*M* = 55.45, *SD* = 28.99) reported significantly higher HPV vaccination intentions than participants with sexual experience in the Self condition (*M* = 43.79, *SD* = 28.31; *b* = 7.02, *t* [388] = 2.78, *p* < 0.01, 95% CI [4.50, 9.54]). Participants without sexual experiences (28.3%) in the Both condition reported similar HPV vaccination intention (*M* = 33.56, *SD* = 28.37) as participants without sexual experiences in the Self condition (*M* = 41.17, *SD* = 31.29; *b* = 3.73, *t* [161] = 0.96, *p* = 0.34, 95% CI [−11.39, 3.92]) (See Figure 2 for more details).

Next, we examined sexual activity (yes, no, omitting “prefer not to say” responses), which also significantly influenced intentions to get vaccinated (*b* = 12.82, *t* [557] = 4.96, *p* < 0.001, 95% CI [7.74, 17.89]). When including sexual activity in the model, participants in the Both condition reported significantly higher HPV vaccination intention compared to participants in the Self condition (Both vs. Self: *b* = 6.81, *t* [551] = 2.47, *p* = 0.01, 95% CI [1.39, 12.24]). With sexual activity in the model, participants in all three intervention conditions reported similar vaccine intentions as participants in the Control condition (Intervention vs. Control: *b* = 0.61, *t* [551] = 0.64, *p* = 0.52, 95% CI [−2.48, 1.27]); and participants in the Self condition reported similar vaccine intentions as participants in the Partner condition (Self vs. Partner: *b* = 2.38, *t* [551] = 0.85, *p* = 0.40, 95% CI [−7.91, 3.15]). Furthermore, current sexual activity did not significantly interact with any of the planned comparisons (*p* > 0.05) and hence did not influence the strength of the effect of intervention type on vaccination intentions.

Lastly, we examined the impact of relationship status on vaccination intentions. There was neither a significant main effect nor interaction of relationship status on vaccination intentions across conditions (at *p* > 0.05).

### 3.4. Attitudes towards Vaccination

Along with relationship and sexual history, we also measured general attitudes regarding vaccines more broadly. Because there is variance in the extent to which individuals are open to getting vaccinated, one possibility is that only individuals who are generally open to getting preventative vaccinations will be impacted by different informational interventions. However, while general attitudes around vaccination significantly predicted HPV vaccination intentions (*b* = 3.16, *t* [575] = 2.71, *p* < 0.01, 95% CI [0.87, 5.46]), they did not significantly interact with intervention conditions across any of the planned comparisons (Interventions vs. Control, *b* = 0.11, *t* [575] = 0.18, *p* = 0.86, 95% CI [−1.35, 1.12]; Both vs. Self, *b* = 0.14, *t* [575] = 0.07, *p* = 0.95, 95% CI [−4.11, 3.83]; Self vs. Partner, *b* = 0.76, *t* [575] = 0.39, *p* = 0.70, 95% CI [−3.12, 4.65]).

### 3.5. Agentic and Communal Values

Finally, we tested whether general endorsement of agentic or communal values influenced vaccine intentions. It is possible that informational interventions describing impacts of HPV infections on others are more effective among people who are generally more communal (or partner-oriented) as compared to people who are generally more agentic (or self-oriented). We found that agentic value significantly predicted vaccination intentions (*b* = 4.10, *t* [575] = 4.10, *p* < 0.001, 95% CI [1.94, 5.50]). However, endorsements of agentic values did not interact with intervention conditions across any of the planned comparisons (Interventions vs. Control: *b* = 0.58, *t* [575] = 1.14, *p* = 0.25, 95% CI [–1.59, 0.42]; Both vs. Self: *b* = 2.11, *t* [575] = 1.45, *p* = 0.15, 95% CI [–0.74, 4.97]; Self vs. Partner: *b* = 1.19, *t* [575] = 0.79, *p* = 0.43, 95% CI [–4.12, 1.75]). Surprisingly, there was no significant effect of endorsement of communal values on vaccination intentions (*b =* 1.44, *t* [575] = 1.76, *p* = 0.08, 95% CI [–0.17, 3.06]), with its interaction with each of the planned comparisons failing to reach significance as well (Interventions vs. Control: *b* = 0.42, *t* [575] = 0.93, *p* = 0.35, 95% CI [–1.31, 0.47]; Both vs. Self: *b* = 2.61, *t* [575] = 1.87, *p* = 0.06, 95% CI [–0.13, 5.35]; Self vs. Partner: *b* = 1.19, *t* [575] = 0.92, *p* = 0.36, 95% CI [–3.72, 1.35]).

## 4. Discussion

The current randomized experiment examined the effect of different informational interventions on promoting HPV vaccination intentions among heterosexual adult men. Overall, when controlling for differences in baseline demographic characteristics, men who received information on how HPV impacts both themselves and their partner reported significantly higher intentions to get vaccinated than when only receiving information on how HPV impacts themselves. Importantly, this effect was influenced by whether men had a prior history of sexual activity. For men who reported having a history of sexual activity, receiving information on how HPV impacts both themselves and their partner significantly boosted their vaccination intentions compared to receiving information only on how HPV impacts themselves. Individuals without a history of sexual activity reported similar intentions to get vaccinated in all intervention conditions and the Control condition.

Interestingly, despite the boost in HPV vaccination intentions among men who were informed about HPV risks for themselves and their partner, no significant differences were detected when providing information about risk for themselves only or for their partner only. While this replicates prior work [11,13], this finding does not fully support either of our initial hypotheses. Because information on both how HPV impacts oneself and one’s partner boosted vaccination intentions, we find some evidence that men can be swayed by partner-oriented information. Nevertheless, the results do not support the notion that, due to reduced conflict with traditional masculine norms, men are more inclined to take preventive health measures to safeguard their partner over themselves. Instead, it seems that providing information on the protective benefits of HPV vaccination for both oneself and one’s partner has a compounding impact. It is possible that this informational intervention was effective because it highlighted the most comprehensive risks of HPV out of the three interventions.

Another important finding is that the control message—which provided general information on HPV and the HPV vaccine without highlighting the benefit of the vaccine for oneself or their partner—performed comparably in influencing vaccine intentions to the informational interventions. This is striking, as the control message provided the fewest reasons to get vaccinated of the four interventions and was also the least tailored to the target population. However, this finding may suggest a possible backfire effect to identity-based informational interventions. Marketing research has found that despite its frequent deployment, identity-based labelling—which either directly or indirectly highlights how a product is intended for a certain population—can inadvertently backfire when the target audience sees that appeal as categorizing them under a single identity [26,27]. Here, it may be the case that when an informational intervention highlights a single facet of their identity—either as a man or person attracted to women—this information may lead men to resent this categorization, thereby rendering the appeal less effective. Thus, while these findings suggest that providing information on the risks of HPV for men and their partners outperforms information on the risks of HPV for men alone—particularly when those men have had a history of sexual activity—it might be fruitful to examine possible backfire effects of this identity-based informational intervention among certain sub-populations of men.

Finally, we found that only a low rate of participants sought further information to book an HPV vaccine appointment during the experiment (1.54%). It may be the case that while the participants had a relatively high intention to get vaccinated in the near future, they may have not necessarily had the time to sign up for a vaccination appointment immediately after completing the study. Additionally, it may be the case that certain populations, such as those found on online survey platforms, may be generally less willing to click external links than they otherwise would be if they had encountered the link in another setting. Both of these possibilities warrant future research.

In sum, these findings have important practical implications for future public health campaigns to promote HPV vaccination uptake. Since adult male populations are often overlooked when examining interventions to boost HPV vaccination despite their far lower vaccination rates, this experiment bridges a gap in the current HPV vaccination literature while offering actionable informational interventions. Additionally, this study samples a more representative demographic beyond that of male college students used in prior studies, making these findings more broadly generalizable. There are possible limitations when considering the implications of this work. First, the current experiment did not directly measure vaccination uptake. However, under the Theory of Planned Health Behavior, expressed intentions are the stronger predictor of behavioral outcomes [28]. Additionally, these findings may not generalize to male populations with higher barriers to immunization such as a higher cost or under-availability of HPV vaccines than currently found in this sample in the United States. Relatedly, because there is variation in the extent to which men prescribe to traditional masculine norms in different populations, which in turn influences the uptake for preventative health behaviors, it will be important to extend this work to different cultural contexts in the future.

## 5. Conclusions

This study tested the effectiveness of different informational interventions on HPV vaccination intentions amongst eligible unvaccinated heterosexual men in the United States. We found that for men with sexual experiences, highlighting how HPV affects themselves and their partner boosted reported vaccination intentions by 10.75 points on a 100-point scale compared to when highlighting information on their personal risks alone. This result demonstrates that combining information about the benefits for oneself and their partner has a compounding impact on HPV vaccination intentions. Thus, this randomized experiment offers valuable guidance for public health officials and policymakers. We demonstrate how a low-cost informational intervention may be implemented to boost vaccination rates amongst a largely under vaccinated population to more effectively combatting HPV transmission.

## Figures and Tables

**Table 1:** Overview of experimental conditions and intervention content.

Condition	*n*	Content	Reference	Word Count
Self	145	HPV-associated male diseases plus HPV general infection and vaccine information. “Protect yourself. Get vaccinated against HPV today”	CDC [1], Saraiya et al., 2015 [22]	325
Partner	144	HPV-associated female diseases plus HPV general infection and vaccine information. “Protect your partner. Get vaccinated against HPV today”	CDC [1], Saraiya et al., 2015 [22]	358
Both	141	HPV-associated male and female diseases plus HPV general infection and vaccine information. “Protect yourself and your partner. Get vaccinated against HPV today”	CDC [1], Saraiya et al., 2015 [22]	467
Control	153	HPV general infection and vaccine information. “Get vaccinated against HPV today”	CDC [1]	231

**Table 2:** Demographic characteristics of the participants (*N* = 583).

Baseline Characteristics	Both		Partner-Oriented		Self-Oriented		Control	
	*n*	%	*n*	%	*n*	%	*n*	%
Gender								
Male	141	100	144	100	145	100	153	100
Age								
18–26	141	100	144	100	145	100	153	100
Ethnicity								
White	71	50	71	49	75	52	76	50
Black or African American	36	26	41	28	36	25	41	27
Hispanic	13	9	13	9	16	11	15	10
American Indian or Alaska Native	0	0	0	0	2	1	1	1
Asian	18	13	13	9	13	9	19	12
Native Hawaiian or Pacific Islander	0	0	2	1	1	1	0	0
Others	3	2	4	3	2	1	1	1
Education								
High School or Less	5	4	8	6	8	6	6	4
High School Graduate	72	51	77	53	87	60	83	54
Associate’s Degree	22	16	17	12	11	8	16	10
Bachelor’s Degree	38	27	33	23	33	23	37	24
Master’s or Professional Degree	2	1	8	6	5	3	10	7
Doctorate Degree	2	1	1	1	1	1	1	1
Occupation								
Full-time	67	48	57	40	67	46	75	49
Part-time	25	18	22	15	30	21	24	16
Start New Job in Next Month	0	0	1	1	3	2	3	2
Unemployed	22	16	30	21	20	14	14	9
Not Gainfully Employed	0	0	2	1	2	1	2	1
Student	25	18	27	19	21	14	31	20
Others	2	1	5	3	2	1	4	3
Income								
Less than USD 9999	16	11	18	13	20	14	16	10
USD 10,000–49,999	37	26	59	41	46	32	54	35
USD 50,000–99,999	61	43	46	32	55	38	42	27
USD 100,000–149,999	18	13	16	11	18	12	22	14
USD 150,000 or more	9	6	5	3	6	4	19	12
Sex History								
Yes	97	69	94	65	95	66	106	69
No	36	26	49	34	42	29	38	25
Prefer Not to Say	8	6	1	1	8	6	9	6
Sexually Active								
Yes	81	57	75	52	79	54	85	56
No	54	38	66	46	57	39	62	41
Prefer Not to Say	6	4	3	2	9	6	6	4
Relationship Status								
Single	80	57	95	66	91	63	90	59
In Relationship	36	26	27	19	40	28	38	25
Recently Ended a Relationship	2	1	3	2	1	1	0	0
Dating	6	4	4	3	3	2	7	5
Engaged	3	2	3	2	1	1	5	3
Married	13	9	11	8	9	6	11	7
Prefer Not to Say	1	1	1	1	0	0	2	1

## Data Availability

The data presented in this study will be available in a publicly available repository on Open Science Framework at https://osf.io/fd6mh/ upon publication.

## Appendix A

**Table A1:** Pre-screening survey.

Category	Pre-Screening Question	Choices
HPV Vaccine History	Have you previously received at least one dose of the human papillomavirus (HPV) vaccine?	No, Yes, Unsure
	Are you currently scheduled to receive an HPV vaccination?	No, Yes, Unsure
General Demographics	What is your sex assigned at birth?	Male, Female, Prefer not to say
	What is your current gender identity?	Female, Male, Transgender Female, Transgender Male, Gender Variant/Non-conforming, Not listed ____, Prefer not to say
	Which of the following best describes your sexual orientation?	Straight/Heterosexual, Homosexual, Bisexual, Asexual, Other, Not listed ___, Prefer not to say
	What is your age?	0–17, 18–26, 27–35, 35–50, 50+, prefer not to say
Others	Do you have a yeast sensitivity or allergy?	No, Yes, Prefer not to say

**Table A2:** Intervention text by conditions.

Condition	Text
Both	**HPV (Human Papillomavirus):** HPV infections are very common. Nearly everyone will get HPV at some point in their lives. Currently, more than 42 million Americans are infected with HPV types that cause disease. About 13 million Americans, including teens, become infected each year. HPV is spread through intimate skin-to-skin contact. You can get HPV by having vaginal, anal, or oral sex with someone who has the virus, even if they do not have signs or symptoms. Most HPV infections (9 out of 10) will go away on their own within 2 years. But sometimes, HPV infections will last longer and can cause some varieties of cancer. Every year in the United States, HPV causes about 36,000 cases of cancer in men and women. **HPV Vaccination Information:** The HPV vaccine provides safe, effective, and long-lasting protection against cancers caused by HPV. The HPV vaccine can prevent over 90% of cancers caused by HPV. More than 135 million doses of the HPV vaccine have been distributed since they were licensed in 2006. Data continue to show the vaccines are safe and effective. People who get the first dose at or after 15 years of age need 3 doses of the HPV vaccine. The recommended three-dose schedule is to get the initial dose, then the second dose 1–2 months later, and finally the third dose 6 months after the initial dose. **By getting the HPV vaccine, you could be protecting yourself from cancers like…** **Anal cancer**: HPV is thought to cause 88.7% of male anus cancer in the United States. **Oropharyngeal (Back of the throat) cancer**: HPV is thought to cause 72.4% of male oropharyngeal cancers in the United States. **Penile cancer**: HPV is thought to cause 66.3% of penile cancers in the United States. According to data from 2015 to 2019, an estimated 47,199 new cases of human papillomavirus (HPV)-associated cancers occurred in the United States each year, including 21,022 among men. **By getting the HPV vaccine, you could be protecting your female partner from cancers like…** **Anal cancer**: HPV is thought to cause 92.5% of female anus cancers in the United States. **Cervical cancer**: HPV is thought to cause 90.6% of cervical cancers in the United States. Vaginal cancer: HPV is thought to cause 75.5% of vaginal cancers in the United States. **Vulval cancer**: HPV is thought to cause 68.8% of vulva cancers in the United States. **Oropharyngeal (Back of the throat) cancer**: HPV is thought to cause 63.3% of female oropharyngeal cancers in the United States. According to data from 2015 to 2019, an estimated 47,199 new cases of human papillomavirus (HPV)-associated cancers occurred in the United States each year, including 26,177 among women. **[Protect yourself and your partner. Get vaccinated against HPV today.]**
Control	**HPV (Human Papillomavirus):** HPV infections are very common. Nearly everyone will get HPV at some point in their lives. Currently, more than 42 million Americans are infected with HPV types that cause disease. About 13 million Americans, including teens, become infected each year. HPV is spread through intimate skin-to-skin contact. You can get HPV by having vaginal, anal, or oral sex with someone who has the virus, even if they do not have signs or symptoms. Most HPV infections (9 out of 10) will go away on their own within 2 years. But sometimes, HPV infections will last longer and can cause some varieties of cancer. Every year in the United States, HPV causes about 36,000 cases of cancer in men and women. **HPV Vaccination Information:** The HPV vaccine provides safe, effective, and long-lasting protection against cancers caused by HPV. The HPV vaccine can prevent over 90% of cancers caused by HPV. More than 135 million doses of the HPV vaccine have been distributed since they were licensed in 2006. Data continue to show the vaccines are safe and effective. People who get the first dose at or after 15 years of age need 3 doses of the HPV vaccine. The recommended three-dose schedule is to get the initial dose, then the second dose 1–2 months later, and finally the third dose 6 months after the initial dose. **[Get vaccinated against HPV today.]**
Other-oriented	**HPV (Human Papillomavirus):** HPV infections are very common. Nearly everyone will get HPV at some point in their lives. Currently, more than 42 million Americans are infected with HPV types that cause disease. About 13 million Americans, including teens, become infected each year. HPV is spread through intimate skin-to-skin contact. You can get HPV by having vaginal, anal, or oral sex with someone who has the virus, even if they do not have signs or symptoms. Most HPV infections (9 out of 10) will go away on their own within 2 years. But sometimes, HPV infections will last longer and can cause some varieties of cancer. Every year in the United States, HPV causes about 36,000 cases of cancer in men and women. **HPV Vaccination Information:** The HPV vaccine provides safe, effective, and long-lasting protection against cancers caused by HPV. The HPV vaccine can prevent over 90% of cancers caused by HPV. More than 135 million doses of the HPV vaccine have been distributed since they were licensed in 2006. Data continue to show the vaccines are safe and effective. People who get the first dose at or after 15 years of age need 3 doses of the HPV vaccine. The recommended three-dose schedule is to get the initial dose, then the second dose 1–2 months later, and finally the third dose 6 months after the initial dose. **By getting the HPV vaccine, you could be protecting your female partner from cancers like…** **Anal cancer**: HPV is thought to cause 92.5% of female anus cancers in the United States. **Cervical cancer**: HPV is thought to cause 90.6% of cervical cancers in the United States. Vaginal cancer: HPV is thought to cause 75.5% of vaginal cancers in the United States. **Vulval cancer**: HPV is thought to cause 68.8% of vulva cancers in the United States. **Oropharyngeal (Back of the throat) cancer**: HPV is thought to cause 63.3% of female oropharyngeal cancers in the United States. According to data from 2015 to 2019, an estimated 47,199 new cases of human papillomavirus (HPV)-associated cancers occurred in the United States each year, including 26,177 among women. **[Protect your partner. Get vaccinated against HPV today.]**
Self-oriented	**HPV (Human Papillomavirus):** HPV infections are very common. Nearly everyone will get HPV at some point in their lives. Currently, more than 42 million Americans are infected with HPV types that cause disease. About 13 million Americans, including teens, become infected each year. HPV is spread through intimate skin-to-skin contact. You can get HPV by having vaginal, anal, or oral sex with someone who has the virus, even if they do not have signs or symptoms. Most HPV infections (9 out of 10) will go away on their own within 2 years. But sometimes, HPV infections will last longer and can cause some varieties of cancer. Every year in the United States, HPV causes about 36,000 cases of cancer in men and women. **HPV Vaccination Information:** The HPV vaccine provides safe, effective, and long-lasting protection against cancers caused by HPV. The HPV vaccine can prevent over 90% of cancers caused by HPV. More than 135 million doses of the HPV vaccine have been distributed since they were licensed in 2006. Data continue to show the vaccines are safe and effective. People who get the first dose at or after 15 years of age need 3 doses of the HPV vaccine. The recommended three-dose schedule is to get the initial dose, then the second dose 1–2 months later, and finally the third dose 6 months after the initial dose. **By getting the HPV vaccine, you could be protecting yourself from cancers like…** **Anal cancer**: HPV is thought to cause 88.7% of male anus cancer in the United States. **Oropharyngeal (Back of the throat) cancer**: HPV is thought to cause 72.4% of male oropharyngeal cancers in the United States. Penile cancer: HPV is thought to cause 66.3% of penile cancers in the United States. According to data from 2015 to 2019, an estimated 47,199 new cases of human papillomavirus (HPV)-associated cancers occurred in the United States each year, including 21,022 among men. **[Protect yourself. Get vaccinated against HPV today.]**

## Appendix B

**Table A3:** Mediator table.

				Descriptive Statistics								*p*-Values for Overall Test and Planned Contrast			
				Both		Control		Other		Self					
	Item Type	Item Text	Scale	*Mean*	*SD*	*Mean*	*SD*	*Mean*	*SD*	*Mean*	*SD*	Overall Test	Control vs. Experimental	Both vs. Self	Self vs. Partner
Attitude	vaccine_ benefit	How beneficial do you think it is for you to get the HPV vaccine?	0 (not at all beneficial) to 100 (extremely beneficial)	65.07	28.96	63.35	28.01	62.88	29.47	61.21	30.20	0.03	0.85	0.09	0.83
	vaccine_ risk	How risky do you think it is for you to get the HPV vaccine?	0 (not at all risky) to 100 (extremely risky) Likert scale	33.21	25.80	33.96	26.88	32.87	28.36	33.19	27.67	0.02	0.28	0.90	0.87
	vaccine_ worried	To what extent are you worried about getting the HPV vaccine?	1 (not at all worried) to 5 (extremely worried)	2.40	1.12	2.22	1.13	2.40	1.21	2.26	1.16	0.02	0.94	0.83	0.26
	HPV_ sideeffect	How serious do you think the possible side effects of the HPV vaccines are for you?	1 (not at all serious) to 5 (extremely serious)	2.47	1.13	2.58	1.26	2.41	1.18	2.41	1.21	0.00	0.09	0.60	0.41
	HPV_ healththreat	How serious of a threat do you believe the HPV vaccine is for your health?	1 (not at all serious) to 5 (extremely serious)	2.33	1.13	2.38	1.25	2.31	1.21	2.35	1.23	0.00	0.14	0.98	0.63
	HPV_ infectionthreat	How serious of a threat do you believe a HPV infection is for your health?	1 (not at all serious) to 5 (extremely serious)	3.22	1.28	3.02	1.17	2.99	1.27	3.15	1.14	0.00	0.65	0.43	0.49
Social Norm	subnorm_ people	Most people who are important to me think that I should get vaccinated against HPV.	1 (completely disagree) to 7 (completely agree)	4.18	1.83	4.22	1.86	3.88	1.89	4.11	1.81	0.09	0.26	0.20	0.06
	subnorm_ expectation	It is expected that I will get vaccinated against HPV.	1 (completely disagree) to 7 (completely agree)	4.28	1.76	4.10	1.88	4.04	1.94	3.91	1.82	0.10	0.55	0.06	0.63
	subnorm_ want	The people in my life whose opinions I value most would want me to get vaccinated against HPV.	1 (completely disagree) to 7 (completely agree)	4.40	1.82	4.44	1.83	4.29	2.01	4.15	1.78	0.07	0.41	0.14	0.88
Perceived Behavioral Control	becontrol_ uptome	Whether I get the HPV vaccine or not is not entirely up to me.	1 (strongly agree) to 7 (strongly disagree)	4.28	2.24	4.05	2.25	4.35	2.27	4.16	2.25	0.93	0.47	0.62	0.91
	becontrol_ control	Getting the HPV vaccine is beyond my control.	1 (strongly agree) to 7 (strongly disagree)	4.94	1.92	4.92	2.05	5.10	2.05	4.82	2.09	0.84	0.68	0.66	0.14
	becontrol_ confidence	I am confident that I can get the HPV vaccine.	1 (strongly agree) to 7 (strongly disagree)	3.50	1.93	3.42	1.98	3.40	2.15	3.41	1.89	0.89	0.88	0.57	0.94
Trust	Infotrust	How much do you trust the information you read about the HPV vaccine?	1 (do not trust at all) to 6 (completely trust)	4.30	1.27	4.33	1.22	4.35	1.30	4.21	1.24	0.80	0.71	0.85	0.50
	Vaxtrust_ safe	Overall, how much do you trust that the HPV vaccine will be safe for you?	1 (do not trust at all) to 6 (completely trust)	4.28	1.24	4.41	1.27	4.31	1.42	4.25	1.36	0.74	0.29	0.96	0.77
	Vaxtrust_ effective	Overall, how much do you trust that the HPV vaccine will be effective?	1 (do not trust at all) to 6 (completely trust)	4.38	1.27	4.36	1.33	4.42	1.29	4.37	1.33	0.98	0.80	0.87	0.71

## Appendix C

Statistics check of the result.
